# The Effect of Alpha Neurofeedback Training with Auditory Feedback on Cognitive Control in Healthy Young Adults: How the Neurological Effects Vary by Stroop Task Difficulty

**DOI:** 10.3390/brainsci16080886

**Published:** 2026-08-20

**Authors:** Nana Matsuo, Kazuma Mikata, Nagomi Okumura, Wataru Inoue, Kyoka Matsui, Takayuki Kodama

**Affiliations:** 1 Faculty of Health Sciences, Kyoto Tachibana University, Kyoto 607-8175, Kyoto, Japan; kodama-t@tachibana-u.ac.jp; 2 Department of Rehabilitation, Hyogo Prefectural Rehabilitation Central Hospital, Kobe 651-2181, Hyogo, Japan; m.kazu0823_m@icloud.com; 3 Rehabilitation Center, Rinku General Medical Center, Izumisano 598-8577, Osaka, Japan; okumura_0517@outlook.jp; 4 Rehabilitation Unit, University Hospital, Kyoto Prefectural University of Medicine, Kyoto 602-8566, Kyoto, Japan; wata016@koto.kpu-m.ac.jp; 5 Department of Rehabilitation, Mibu Oji Hospital, Kyoto 600-882, Kyoto, Japan; nimi930kyo24@outlook.jp

**Keywords:** cognitive control function, New Stroop Test I, cognitive load, alpha neurofeedback, task difficulty

## Abstract

**Highlights:**

**What are the main findings?**

- A single-session auditory alpha neurofeedback (αNFB) intervention was associated with an increased α/β ratio during low cognitive load task performance, whereas no significant neurophysiological effects were observed during high cognitive load task performance.
- Neurophysiological changes were observed without corresponding differences in behavioral task performance.

**What are the implications of the main findings?**

- The observed pattern is consistent with the possibility that neurophysiological responses to auditory αNFB may differ according to cognitive load, although this finding should be interpreted cautiously given the exploratory nature of the study.
- The findings provide preliminary evidence that auditory αNFB may influence neurophysiological responses during some cognitive-task conditions, warranting further investigation in larger studies.

**Abstract:**

**Background:** Cognitive control is the ability to adapt to varying cognitive demands. Alpha neurofeedback (α-NFB) training is reported to affect neural activity, but little is known about whether neurophysiological responses to auditory α-NFB differ across cognitive tasks with different cognitive load. This exploratory pilot study examined the neurophysiological effects of a single session of auditory α-NFB during cognitive tasks with different cognitive loads. **Method:** Fifteen healthy young adults participated in the study. Participants completed Tasks 1 and 3 of the New Stroop Test I before and after the intervention. Neural oscillations and task-performance measures were assessed before and after the intervention. Pre–post changes were compared between the intervention and control groups. **Results:** For Task 1, the intervention group exhibited a significantly greater pre–post intervention change in the α/β ratio than the control group. However, the increase in alpha oscillatory power did not remain statistically significant after Holm correction for multiple comparisons. For Task 3, no significant between-group differences were observed in any neurophysiological or behavioral measure. **Conclusions:** These exploratory findings suggest that auditory αNFB may influence neurophysiological responses during Task 1, whereas no significant neurophysiological effects were observed during Task 3. This pattern is consistent with the possibility that neurophysiological responses to auditory αNFB may differ according to cognitive load; however, this interpretation remains preliminary and requires confirmation in larger studies. However, no corresponding differences in task performance were observed, and the findings should be interpreted as preliminary and hypothesis-generating.

## 1. Introduction

In everyday life, people must accomplish different tasks efficiently, switching smoothly between them. Task switching entails cognitive load, resulting in switch costs [1]. Switch costs represent the additional response time and cognitive load incurred when switching from one task to another [2]. Thus, controlling such switch costs is an important part of cognitive control [1,3]. As people age, they experience increased switching costs and diminished cognitive control [4]. Consequently, they become less efficient in daily tasks and social interactions, directly impacting their quality of life. A number of studies have investigated the mechanism by which switch costs arise [5,6]. Among these, Gajewski et al. (2009) [5] noted the need to reconstruct preparedness for the upcoming task. Thus, they proposed the reconstruction processing time hypothesis, which states that switch costs represent the extra time spent in cognitive control for such [5]. If cognitive control can be regulated extrinsically, it may be possible to accomplish tasks more efficiently while responding to situations involving diverse cognitive loads.

Among potential extrinsic approaches to improving cognitive function, one study reported the effectiveness of long-term neurofeedback training in controlling alpha oscillations [7]. In alpha neurofeedback training (α-NFB), the person receives real-time sensory feedback (audio, visual, or tactile) when their neural oscillations fall within the alpha-frequency band, thereby helping them autonomously control their neural activity [8]. One study subjected healthy young adults to a short-term α-NFB intervention with tactile feedback. The intervention improved participants’ cognitive control and was reported to be therapeutically effective [7]. However, relying on tactile feedback imposes a limitation in that it restricts participants’ hand movements, precluding multitasking. Auditory feedback, by comparison, would impose less of a strain on task performance. Thus, αNFB with auditory feedback (“auditory αNFB”) would be compatible with a greater range of tasks and thus more practical. Most studies investigating αNFB have employed visual feedback modalities, and evidence regarding auditory αNFB remains comparatively limited. Recent studies have suggested that auditory neurofeedback, particularly music-based auditory feedback, may successfully facilitate alpha-wave modulation while reducing the attentional demands associated with continuously monitoring visual feedback displays [9,10]. Auditory feedback may therefore be advantageous in situations in which participants are simultaneously engaged in cognitive tasks or other activities requiring visual attention. Nevertheless, despite these potential advantages, few studies have examined the neurophysiological effects of auditory αNFB during cognitive control tasks, and it remains unclear whether its effects differ according to cognitive-load conditions. Therefore, the novelty of the present study lies in examining the immediate neurophysiological effects of a single-session auditory αNFB intervention during tasks involving different levels of cognitive load. Despite this possibility, little is known about how the effect of auditory αNFB varies by cognitive-load condition. To address this gap in the literature, the present study subjected healthy young adults to auditory αNFB to investigate the effect of a single session of αNFB upon cognitive control function. This study was conducted as an exploratory pilot study, with approximately 15 participants planned from the outset to obtain preliminary evidence regarding the neurophysiological effects of a single session of auditory αNFB under different cognitive-load conditions. Accordingly, two hypotheses were formulated. Hypothesis 1 was that a single session of auditory αNFB would induce measurable neurophysiological changes during cognitive task performance. Hypothesis 2 was that these neurophysiological effects would be more pronounced during a task involving relatively low cognitive load than during a task involving higher cognitive load. Given the exploratory nature of this pilot study, no specific directional hypothesis was established regarding behavioral performance outcomes.

## 2. Materials and Methods

### 2.1. Participants

Participants were recruited using a convenience sampling method from the student body of first author’s university. The study participants were fifteen healthy young adults, with seven men and eight women, with a mean age of 21. All participants were university students. Participants were randomly assigned to either the intervention group or the control group using a sex-stratified randomization procedure. Specifically, male and female participants were randomized separately by drawing lots to ensure a balanced sex distribution between groups. The allocation was concealed until the time of assignment. Participation in the study was voluntary, and participants were excluded if they had a medical condition or were taking medication. Handedness was not assessed in the present study. The target sample size was set at approximately 15 participants at the outset of the study because the study was designed as an exploratory pilot investigation to obtain preliminary evidence regarding the neurophysiological effects of a single session of auditory αNFB under different cognitive-load conditions; no a priori power calculation was performed. Written informed consent was obtained from the participants following a briefing about the study’s methods and purpose.

### 2.2. Method

**Figure 1:**
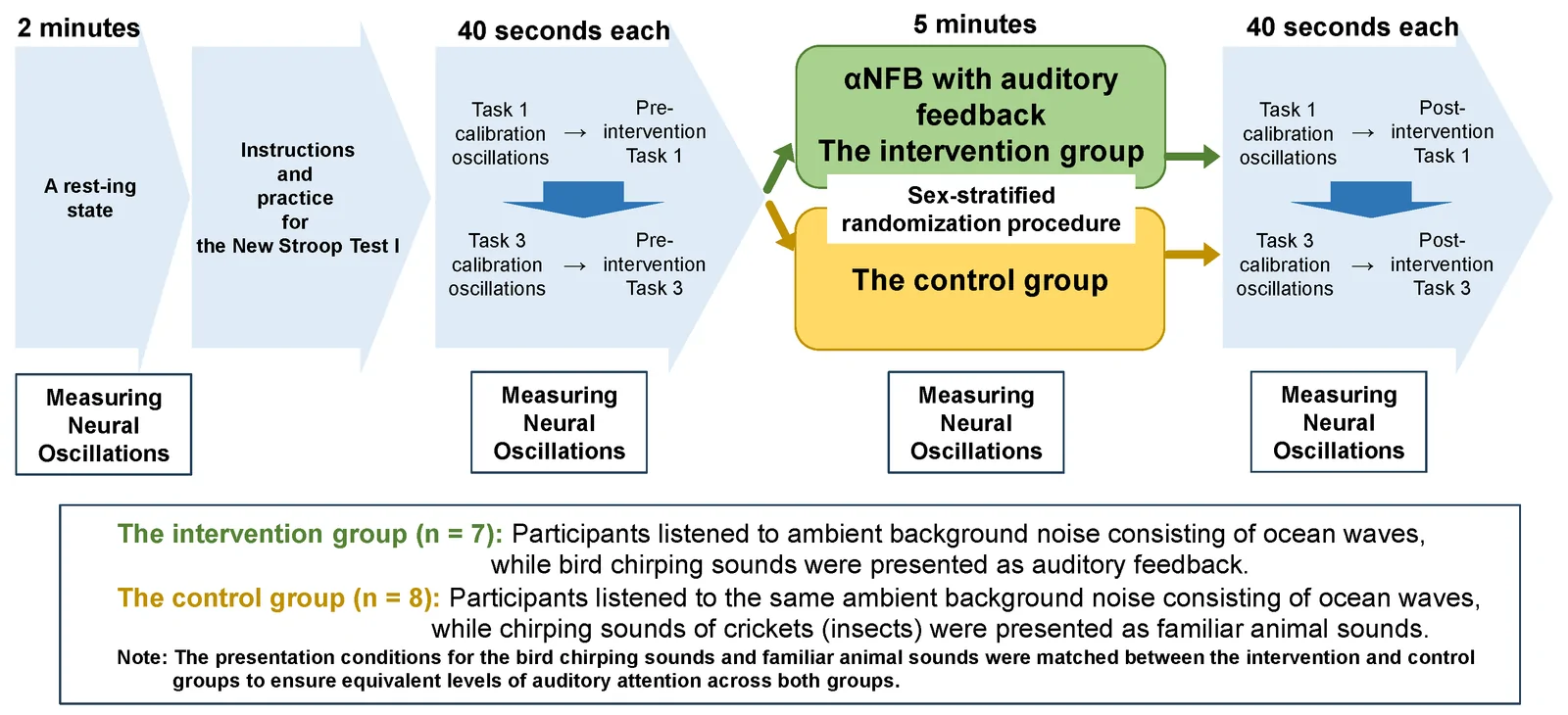
Study protocol.

The study protocol is presented in Figure 1. The participants were assigned identification codes and allocated to the intervention group or the control group using the sex-stratified randomization procedure described in Section 2.1, thereby ensuring a balanced distribution of male and female participants across both conditions. The intervention group consisted of three men and four women, while the control group consisted of four men and four women. Participants were instructed to refrain from eating for one hour prior to the start of the experiment to avoid drowsiness. They were also instructed to abstain from caffeine intake and avoid excessive exercise before the experiment. All participants, regardless of group, had their neural oscillations measured for 2 min in a resting state and then undertook two cognitive tasks (Tasks 1 and 3 of the New Stroop Test I, described below) twice, once pre-intervention and once post-intervention. Following the pre-test, the intervention group underwent one session of auditory αNFB. Neural oscillations were recorded during each task. Neural oscillations were then compared between the pre- and post-intervention tasks.

#### 2.2.1. Cognitive Tasks

The two cognitive tasks were from the group edition of Toyo Physical’s New Stroop Test I (Toyo Physical Co., Ltd., Fukuoka, Japan) [11], which measures attentional function in the frontal lobe. The New Stroop Test I consists of four tasks, each involving a series of five color words (such as the words “red” and “blue”) written in hiragana script, along with a set of five corresponding colors. Of these four tasks, Tasks 1 and 3 were used in the present study. Tasks 1 and 3 were used to impose a load on participants’ attentional control. Task 1 requires a person to indicate the color that matches the word, while Task 3 requires a person to indicate the word that matches the color. These two tasks were chosen because they involve no Stroop interference and because their difficulty varies, whereas the other two tasks (Tasks 2 and 4) require, upon stimulus presentation, selective attentional control, representational switching, and Stroop interference [11].

In Task 1, participants were presented with a sequence of color words, each printed in black ink. For each word presented, participants were required to select from among the set of five colors, presented on their right, the color that matches the meaning of the word, and to indicate their selection by marking the color. In Task 3, participants were presented with a sequence of colors. For each color presented, they were required to select the corresponding color’s word and to indicate their selection by marking it. Each task was undertaken on a single sheet of paper, with 40 items arranged in 20 rows and 2 columns. The task was administered over a period of 40 s, in accordance with the standard administration procedure for the New Stroop Test I [11]. If a participant completed all the items within the time limit, their remaining time was recorded.

#### 2.2.2. Measuring Neural Oscillations

Neural oscillations were measured using an electroencephalogram (EEG; Brain Pro FM—929, Futek Electronics Co., Ltd., Yokohama, Japan). To ensure reliable data, the EEG was designed to automatically pause recording whenever it detected artifacts, meaning signals originating from sources other than the brain (e.g., sources from facial muscles or eye movements). The EEG data were analyzed using dedicated software (Brain Pro Analyzer Plus FS—AP, Futek Electronics Co., Ltd., Yokohama, Japan). Electrodes were positioned on the scalp according to the international 10–20 system for electrode positioning. A measuring electrode was positioned at Fp2 (right frontopolar region), an earth electrode was positioned at Fp1 (left frontal lobe), and a reference electrode was positioned at A1 (left ear lobe). The electrode configuration was determined according to the specifications of the EEG device used in this study. The device was configured for single-channel EEG recording from the frontal region, and Fp2 was therefore used as the measurement electrode. According to the manufacturer’s specifications, Fp1 and A1 were designated as the earth and reference electrode sites, respectively, in this configuration. The EEG recordings were processed using dedicated software (Brain Pro Analyzer Plus FS—AP) to generate waveforms at a sampling frequency of 1024 Hz. The power spectral density was estimated in 0.5 Hz increments between 3.0 Hz and 30.0 Hz using a fast Fourier transform with a rectangular window function. Signals below 3.0 Hz were removed from the analysis because they were deemed artifacts (assumed to be ultra-low-frequency signals caused by blinking or eye movements). Similarly, within each 1 s segment, any signal with an amplitude of 20 μV or greater at 3.0 Hz was also deemed an artifact and excluded [8]. This criterion was determined based on the artifact removal settings of the specialized software used for EEG analysis. As in [12], the following neurophysiological metrics were measured during the tasks: oscillatory power within the theta-frequency band (4–7.5 Hz: “θ oscillatory power”), oscillatory power within the alpha-frequency band (8–12 Hz: “α oscillatory power”), and oscillatory power within the beta-frequency band (12.5–30 Hz: “β oscillatory power”). All three metrics were measured as µV^2^.

The recorded values for these three neurophysiological metrics were converted to natural logarithms to normalize their distributions. To accommodate zero values in the dataset, a constant of 0.001 was added to the data prior to the transformation. To account for inter-individual variation in oscillatory power, the neurophysiological values recorded when participants moved (“calibration oscillations”) were subtracted, yielding the neurophysiological values associated with the cognitive task (“net oscillations”) [13]. To examine associations between α oscillatory power and β oscillatory power, the former was divided by the latter to produce the ratio α/β [14,15]. This ratio was named “relaxation rate,” in that it represented oscillations during a relaxed state [16]. The size of the intervention’s effect was defined as the pre–post intervention difference in a given neurophysiological metric (net α oscillatory power, net β oscillatory power, or relaxation rate) for a given cognitive task (Task 1 or 3).

#### 2.2.3. Intervention (αNFB)

The intervention group received auditory αNFB (real-time auditory feedback of alpha oscillations in the frontal lobe). The feedback was presented using the same hardware and software used to measure neural oscillations. The auditory feedback was a sound effect of a bird chirping. During the αNFB intervention, EEG signals were processed in consecutive, non-overlapping 1 s segments. Alpha-frequency band amplitude value was calculated for each 1 s segment and updated once per second (i.e., 0–1 s, 1–2 s, 2–3 s, etc.). Auditory feedback was provided using a threshold-based procedure rather than being continuously proportional to alpha-wave amplitude. Specifically, a bird-chirping sound was presented when the alpha-frequency band amplitude value exceeded the predefined threshold of 4.5 μV, whereas no bird-chirping sound was presented when the amplitude did not exceed this threshold. The sound effect was activated whenever amplitude value within the alpha-frequency band exceeded a certain threshold. That is, the intervention group would hear a bird chirping when alpha frequency band amplitude value exceeded the threshold. The threshold was set at 4.5 μV, based on the available hardware settings and a prior study in which alpha-frequency oscillations of about 4.75 μV were recorded at the start of NFB in participants aged in their 20s (similar to those in this study) [17]. A fixed threshold of 4.5 μV was adopted based on normative resting-state alpha-amplitude values reported in healthy young adults in a previous study. Because the purpose of this exploratory pilot study was to examine the feasibility and preliminary neurophysiological effects of auditory αNFB under standardized conditions, the same threshold was applied to all participants rather than using individually calibrated thresholds. No participant in the intervention group reported feeling unpleasant after hearing the chirping. The intervention consisted of a single five-minute session, a duration based on the literature. Indeed, Nawaz et al. (2022) reported that the effect on alpha oscillations is most pronounced during the first five minutes, meaning five minutes is an optimal duration for a session [10]. Further, Zhou et al. (2024) reported that a five-minute αNFB session improved the quantity and precision/accuracy of visuospatial working memory [18].

#### 2.2.4. Measuring Procedure

In accordance with the study protocol, each task was preceded by a practice session consisting of ten question items. The practice sessions were prepared separately from the main tasks and were designed to familiarize participants with the task’s format prior to commencing the actual task. The practice session was conducted solely to familiarize participants with the New Stroop Test I. Auditory αNFB was not provided during this session; therefore, no neurofeedback timing or threshold parameters were applied prior to the pretest assessment. After the practice task and before Task 1, participants were instructed to make marks on a sheet of paper—which was the same size of the Task 1 sheet —that contained only rectangular marking areas, corresponding to the answer areas of Task 1. The purpose of this step was not disclosed to the participants. This process was undertaken to subtract the calibration oscillations. Next, the Task 1 sheet was provided, and the participants completed the pre-intervention iteration of Task 1. During pre-intervention Task 1, the calibration oscillations were recorded along with the mid-task oscillatory power values. Participants were told to remain as still as possible during the task and to move only their writing hand. They were also told that, if they indicated the wrong answer by mistake, they should continue without correcting the mistake. After completing pre-intervention Task 1, participants undertook pre-intervention Task 3. This task followed the same procedure as Task 1.

After completing pre-intervention Tasks 1 and 3, the intervention group underwent a five-minute auditory αNFB session. During the intervention, participants listened to ambient background noise of ocean waves. When the participant’s brain produced alpha oscillations, the bird chirping sound effect was presented. Control group participants underwent a five-minute αNFB session without auditory feedback. They were presented with ambient background noise consisting of waves and chirping of crickets (insects)—as a familiar animal sound—for a duration of five minutes. For the control group, the cricket sounds were played at the same frequency as the bird sounds used for the intervention group to ensure equivalent levels of auditory exposure across both groups. No participant in the control group reported feeling unpleasant after hearing the cricket chirping. After this intervention, the participants undertook post-intervention Tasks 1 and 3. The procedure for these tasks was the same as that for pre-intervention Tasks 1 and 3. As before, participants’ neural oscillations were measured. To minimize practice effect, the tasks administered before and after the intervention followed the same format as Tasks 1 and 3, but used sheets with different questions. Due to the nature of the intervention, researchers were not blinded to group assignment during EEG measurements. In addition, the personnel who scored the Stroop task responses and processed the EEG data, including artifact rejection, were not blinded to group assignment. Therefore, the possibility of measurement bias cannot be completely excluded.

### 2.3. Statistical Analysis

Pre–post intervention differences in oscillation metrics were subjected to a between-group comparison. Normality was tested using the Shapiro–Wilk test. Because the sample size was small and normality could not be assumed for all variables, between-group comparisons were conducted using the Mann–Whitney U test. The assumptions of the Mann–Whitney U test were considered satisfied because the intervention and control groups consisted of independent observations, each participant contributed only one observation to each analysis, and the outcome variables were measured on continuous scales. Pre–post intervention differences in task performance (completed answers, correct answers, incorrect answers, and response time) were likewise subjected to the Mann–Whitney U test. For EEG artifact exclusion rates and the remaining duration of EEG data after artifact removal, between-group comparisons were also conducted using the Mann–Whitney U test. To control for multiple comparisons, the Holm correction was applied separately to the neurophysiological and behavioral outcomes. For the neurophysiological outcomes, all eight comparisons across Task 1 and Task 3 were treated as a single family and corrected together. Likewise, for the behavioral outcomes, all eight comparisons across Task 1 and Task 3 were treated as a separate family and corrected together. To compare the difficulty between Tasks 1 and 3, the Wilcoxon *t*-test was used to compare all participants’ response times (in seconds) between pre-intervention Task 1 and pre-intervention Task 3. For neurophysiological and task-performance outcomes, 95% confidence intervals (CIs) for between-group differences were calculated using the Hodges–Lehmann estimator of the median difference, as provided by the independent-samples Mann–Whitney U test procedure in SPSS. Statistical data were computed using SPSS version 30.0 (IBM Corp., Armonk, NY, USA). The significance level was set at 5%.

## 3. Results

### 3.1. Baseline Characteristics

Table 1 presents the baseline characteristics of the intervention and control groups. No statistically significant between-group differences were observed in the baseline characteristics.

### 3.2. EEG Artifact Rejection Rate and Remaining Data Duration

Table 2 shows the epoch rejection rates due to artifacts and the duration of remaining EEG data after rejection. Significant between-group differences were identified for the artifact rejection rate in Task 1 post-intervention and for the remaining duration of EEG data in Task 1 pre-intervention. Specifically, the artifact rejection rate in Task 1 post-intervention was significantly higher in the intervention group than in the control group (*p* = 0.01, r = 0.66). In addition, the remaining duration of EEG data in Task 1 pre-intervention was significantly longer in the intervention group than in the control group (*p* = 0.004, r = 0.70). No significant between-group differences were observed for the remaining measures.

### 3.3. Neural Oscillations

Table 3 shows the recorded data items and the analysis results. Regarding Task 1, α oscillatory power was significantly greater in the intervention group than in the control group before correction for multiple comparisons (*p* = 0.01, r = 0.72), but this effect was no longer significant after Holm correction (adjusted *p* = 0.07). By contrast, the α/β ratio was significantly greater in the intervention group than in the control group (*p* = 0.003, r = 0.78) and remained significant after Holm correction (adjusted *p* = 0.02).

For Task 3, no significant difference was observed between the intervention and control groups.

**Figure 2:**
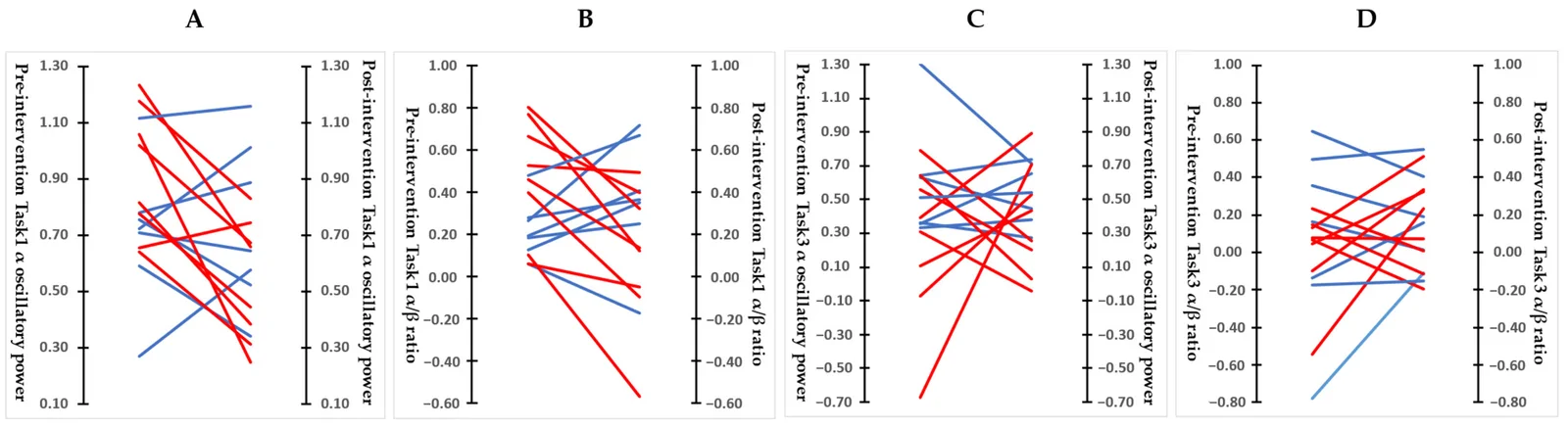
Individual pre–post values of neurophysiological measures. (**A**) α oscillatory power during Task 1. (**B**) The α/β ratio during Task 1. (**C**) α oscillatory power during Task 3. (**D**) The α/β ratio during Task 3. Note: Each line represents an individual participant. Blue lines indicate the intervention group, and red lines indicate the control group.

Figure 2 through 5 show paired plots of alpha oscillatory power and the α/β ratio for all participants for Task 1 and Task 3.

### 3.4. Task Performance

Table 4 presents the results regarding task performance metrics, namely, number of answered questions, number of correct answers, number of incorrect answers, and response time. No inter-group differences were observed in these metrics (see Table 4).

Regardless of group (intervention or control), response time was significantly higher in the first iteration of Task 3 (39.71 ± 0.73) than it was in the first iteration of Task 1 (33.47 ± 4.79) (*p* = 0.004, r = 0.75). When asked verbally about difficulty level, every participant replied that they found Task 3 harder than Task 1.

Regarding task performance, comparisons between groups revealed no significant differences between the intervention and control groups in terms of the number of responses, correct answers, incorrect answers, or reaction times for either Task 1 or Task 3 (see Table 5).

## 4. Discussion

We examined the effect of a single session of auditory αNFB on young adults’ ability to exert cognitive control during cognitive tasks on the New Stroop Test I. The results suggested that the intervention was associated with neurophysiological changes during the relatively easy Task 1. However, because no formal statistical comparison of intervention effects between Tasks 1 and 3 was performed, caution is warranted when interpreting these findings as evidence of task-difficulty-dependent effects.

After Holm correction for multiple comparisons, the increase in α oscillatory power no longer reached statistical significance (adjusted *p* = 0.07), whereas the increase in the α/β ratio remained significant (adjusted *p* = 0.02). Therefore, the neurophysiological effect observed during Task 1 is more robustly reflected by the α/β ratio than by α oscillatory power alone. Indeed, a prior study reported that, in a virtual-reality stress paradigm, the α/β ratio was negatively correlated with stress levels, with higher ratios being observed under less stressful conditions [14]. Another study reported that αNFB interventions reduce cognitive load during cognitive tasks, such as mental rotation and n-back tests [7]. However, these studies employed substantially different experimental paradigms, including virtual-reality stress induction, mental rotation, and n-back tasks, as well as different neurofeedback modalities. Therefore, comparisons with the present findings should be regarded as indirect and interpreted with caution. Considering this research, the observed increase in the α/β ratio, together with the tendency toward increased α oscillatory power, may reflect changes in neurophysiological state during task performance. One possible interpretation is that these changes were associated with reduced cognitive load; however, alternative interpretations cannot be excluded. If the observed neurophysiological changes were associated with reduced cognitive load, they may, in turn, have influenced the process of controlling attention, given that switch costs are associated with the cognitive load entailed by controlling attention [2]. However, increased alpha oscillatory power does not necessarily reflect reduced cognitive load alone. Recent evidence suggests that alpha activity is associated with inhibitory control, the suppression of distracting information, attentional gating, and internally directed cognitive processes [19,20,21]. Therefore, the present findings may reflect changes in attentional allocation, inhibitory processing, and other neurophysiological processes, with reduced cognitive load representing only one possible interpretation.

As for the performance metrics for Task 1, the pre–post intervention difference did not vary between the two groups. One possible explanation for the lack of an inter-group difference is the neural efficiency hypothesis, although this interpretation remains speculative and cannot be confirmed from the present data alone. Consistent with this hypothesis, one study reported that participants, despite showing no clear differences in task performance, differed in the efficiency of cognitive processing, as reflected in neural oscillation patterns [22]. Another study found that alpha oscillations were associated with the ability to resist distracting information and distribute cognitive resources efficiently [23]. Thus, the significant increase in the α/β ratio, together with the nonsignificant trend toward increased α oscillatory power, may have reflected changes in neurophysiological activity during task performance that were not translated into measurable behavioral improvements. Although this pattern is consistent with the neural efficiency hypothesis, alternative explanations, including limited sensitivity of the behavioral measures and potential ceiling effects, cannot be excluded. One study compared processing performance between two tasks in the Stroop Col-or-Word Test, one in which participants had to find the color to match the word, and the other in which they had to find the word to match the color; the researchers reported that processing performance was better in the former task [24]. Given that the present study was conducted on healthy young adults, participants may have found Task 1 (finding the color that matches the word) relatively easy to complete with or without the intervention, which would explain why the intervention had no discernible effect on performance. In other words, a ceiling effect may have occurred [25], which would prevent any clear pre–post intervention difference from reflecting as an improvement in the test performance, despite changes in neural state. The test performance metrics we used (number of answered questions, number of correct answers, number of incorrect answers, and response time), while effective for measuring performance in cognitive tasks in general, may have been less effective in capturing subtle pre–post intervention differences in cognitive control function. In addition, the response-time measure may have been subject to a ceiling effect because each task was administered within a fixed 40 s period according to the standard procedure of the New Stroop Test I. Notably, response times in Task 3 were clustered near this upper limit, suggesting that the measure may have had limited sensitivity to detect subtle intervention-related changes. Therefore, the absence of significant behavioral differences should be interpreted cautiously, as behavioral changes may not have been fully captured by the response-time measure. One study reported that such differences may be too subtle for the Stroop Color-Word Test to capture, and that the effect of such an intervention is unlikely to materialize as a statistically significant inter-group difference [26]. Another report suggested that neural activity is better measured by continuous metrics that reflect sensitivity rather than by discrete behavioral metrics, such as the number of correct answers in the Easy Stroop Task [27]. Thus, in the present study, pre–post intervention changes in neurophysiological values (neural oscillations) did not correspond to pre–post intervention changes in behavioral values (task performance); the neurophysiological effects of the intervention were not immediately replicated at the behavioral level.

Regarding Task 3, there were no pre–post intervention differences between groups in any of the neural oscillation metrics. One reason might be that Task 3 entailed a higher cognitive load. The literature on the Stroop Color-Word Test suggests that tasks requiring participants to select a word corresponding to a presented color entail more complex processing and greater cognitive load than tasks requiring participants to select a color corresponding to a presented word [24,28]. Consistent with this framework, Task 3 of the New Stroop Test I requires participants to indicate the word that matches a presented color, whereas Task 1 requires participants to indicate the color that matches a presented word. Another study on the Stroop Color-Word Test found that performance (correct answers and response time) was worse in Task 3, regardless of participant age [29]. These findings are consistent with the performance that we observed in the New Stroop Test I for groups. In the present study, we found that response times were significantly longer for Task 3. We also obtained participant feedback, which revealed that every participant found Task 3 harder than Task 1. These findings suggest that Task 3 entailed a greater cognitive load than Task 1. Regarding the relationship between cognitive load and oscillatory power, one study reported that alpha oscillatory power is lower during tasks that participants subjectively experience as hard [30]. Thus, the absence of significant neurophysiological effects in Task 3 may be consistent with the possibility that higher cognitive load influenced the response to the intervention. However, the present study was not designed to directly test whether intervention effects differed significantly between task difficulty levels.

We previously noted a lack of synchronicity between the oscillatory power metrics and behavioral metrics. Although this disconnect may be consistent with the neural efficiency hypothesis, it may also reflect the limited sensitivity of the behavioral measures, including possible ceiling effects associated with the task design. The neurophysiological effect of the intervention during Task 1 was most clearly captured by the α/β ratio metric, while the increase in α oscillatory power did not remain statistically significant after correction for multiple comparisons. In contrast, no significant between-group differences were observed in the neurophysiological measures during Task 3, which entailed a greater cognitive load. Thus, the intervention was associated with a physiological effect during Task 1, whereas no significant neurophysiological effects were observed during Task 3. This pattern is consistent with the possibility that neurophysiological responses to auditory αNFB may differ according to cognitive load; however, the present study did not directly test differences in intervention effects between task conditions. Therefore, conclusions regarding task-difficulty-dependent effects should be considered preliminary. Thus, the intervention produced a physiological effect, albeit one that varied by task difficulty, and this effect was not accompanied by a commensurate improvement in behavioral performance. However, the auditory stimuli differed between the intervention and control groups. Although both groups received auditory stimulation during the experimental procedure, the bird-chirping sound used for the intervention group and the cricket sound used for the control group may have differed in semantic meaning, emotional valence, and ecological familiarity. These differences may have influenced participants’ attention or arousal independently of the neurofeedback process and therefore may have contributed to the observed physiological differences. Thus, the present design cannot completely distinguish the effects of auditory αNFB from potential effects attributable to differences in the auditory stimuli themselves. Previous sham-controlled neurofeedback studies have used control conditions in which the sensory feedback was matched between groups while the contingency between the participant’s own brain activity and the feedback was removed, thereby allowing the specific effects of neurofeedback to be distinguished from nonspecific effects of the feedback experience [31]. A sham neurofeedback condition using an acoustically matched stimulus that is not contingent on participants’ alpha activity would therefore provide a stronger control for nonspecific effects of auditory stimulation [30]. Future studies should consider such a sham-controlled design to more specifically determine the neurophysiological effects attributable to auditory αNFB.

### Limitations

This study had the hallmarks of a pilot study, as it was conducted with a small sample of 15 participants. The target sample size of approximately 15 participants was determined a priori based on the exploratory and preliminary nature of the study rather than on a formal a priori power calculation. As such, the results should be considered as exploratory findings. In addition, neural oscillations were recorded using a single-channel EEG system with the measurement electrode positioned at Fp2, in accordance with the manufacturer’s specifications. Although artifact detection was performed during data acquisition and artifact removal during offline analysis, this process differed from corrections based on Independent Component Analysis (ICA) or electrooculography (EOG); thus, it does not guarantee the complete elimination of ocular artifacts. Furthermore, given that the Fp2 site is susceptible to artifacts caused by eye movements and facial muscle activity, the complete exclusion of such contamination cannot be assured. Furthermore, because neural oscillations were recorded from a single frontopolar site, the present findings should be interpreted as reflecting activity at Fp2 rather than frontal cortical activity as a whole. Future studies using multichannel EEG systems with broader electrode coverage are warranted to confirm the present findings.

A further limitation concerns differences in EEG data quality between groups. Significant between-group differences were observed in the artifact rejection rate for Task 1 post-intervention and in the remaining duration of EEG data after artifact removal for Task 1 pre-intervention. Because the principal neurophysiological finding was also observed in Task 1, it cannot be ruled out that these differences affected spectral power estimation. A sensitivity analysis using a standardized duration of artifact-free EEG data was not feasible retrospectively. Accordingly, the present findings should be interpreted with appropriate caution. Furthermore, the examiners involved in electroencephalography (EEG) processing and artifact removal were not blinded to group assignment. Although artifact processing was conducted according to a predetermined procedure, the possibility that information regarding group assignment influenced the data processing cannot be entirely ruled out. Another limitation concerns some implementation-specific parameters of the neurofeedback system, including the feedback update rate, the latency between EEG acquisition and auditory feedback, and the actual frequency of auditory feedback presentation, were not recorded during the experiment and could not be confirmed retrospectively. This should be considered when interpreting the reproducibility of the neurofeedback protocol. Another limitation concerns the use of a fixed alpha-amplitude threshold (4.5 μV) for all participants during the auditory αNFB intervention. Given the substantial inter-individual variability in alpha activity, the amount of auditory feedback received may have differed among participants. Because alpha-amplitude values during the neurofeedback sessions were not recorded, participants’ positions relative to the threshold could not be confirmed. Consequently, the observed effects may have been influenced by individual differences in baseline alpha activity. Furthermore, because the frequency of auditory feedback presentation and changes in alpha activity during the neurofeedback session were not recorded, it was not possible to determine whether participants successfully learned to modulate their alpha activity or engaged in effective self-regulation during the intervention. Accordingly, the present study should be interpreted as evaluating the effects of a single-session auditory neurofeedback procedure rather than demonstrating neurofeedback learning. Another limitation is that the alpha/beta ratio used as the “relaxation rate” in this study should be interpreted with caution, as the alpha/beta ratio is not a universally established or validated measure of relaxation, and evidence supporting its use as a specific index of relaxation remains limited. The study sample was limited to healthy young adults, so further research is necessary to determine whether the results are applicable to older adults or individuals with medical conditions. Further, this study examined only the immediate effect of a single session of the intervention in an experimental setting (using the Stroop test), which limited the external validity of the findings (i.e., the validity of applying the findings to cognitive load scenarios experienced in real life). Another limitation is that Task 1 was always performed before Task 3, and task order was not counterbalanced. Therefore, differences between the two tasks may have been influenced by order effects, including practice and fatigue, in addition to differences in cognitive load. Future studies should employ counterbalanced task orders to minimize this potential bias. We controlled for the attentional effect of the auditory feedback by presenting the same ambient background noise (sound of waves) to both groups of participants and by presenting a bird chirping sound to the intervention group participants and an animal noise to the control group. This attempt to control the attentional effect of auditory feedback may have resulted in attentional arousal and semantic processing varying across types of auditory stimuli. A future study should include stricter measures to control for any confounding effect that may result from the content of the auditory feedback. In the interests of statistical power, a future study should use a larger sample size to confirm whether the oscillatory power metrics we used in our study are robustly associated with cognitive load. It should also use a wider range of cognitive tasks and difficulty levels to assess whether the oscillatory power metrics that we used serve as consistent, objective measures, including measures of their replicability and clinical applicability. Such future research will contribute to an integrated understanding of cognitive function and help establish a clinically practical form of αNFB.

## 5. Conclusions

In this study, we conducted an exploratory pilot investigation to examine the effects of a single session of auditory αNFB. The results showed that the intervention group exhibited a greater pre-to-post change in the alpha/beta ratio than the control group during Task 1, whereas no significant between-group difference was observed during Task 3. These physiological findings were not accompanied by between-group differences in task performance. Thus, the observed physiological changes were not accompanied by measurable behavioral benefits, and the present findings do not provide evidence that auditory αNFB improved cognitive control. Given the small sample size and the exploratory nature of this study, these results should not be interpreted as confirmatory but rather as preliminary and hypothesis-generating. While the observed pattern may be consistent with the possibility that neurophysiological responses to auditory αNFB differ according to cognitive load or task context, this study does not demonstrate that auditory αNFB enhances cognitive control or task performance. Furthermore, because the control condition did not isolate contingent neurofeedback effects from nonspecific auditory-stimulation effects, the observed increase in the α/β ratio should be interpreted with caution. Although the present pilot study suggests that auditory αNFB can be implemented during cognitive control tasks, the findings should be interpreted solely as a preliminary confirmation of feasibility.

## Figures and Tables

**Table 1:** Baseline characteristics of the intervention and control groups.

		The Intervention Group (*n* = 7)	The Control Group (*n* = 8)
Age (years)		21	21
Sex (male/female)		3/4	4/4
Pre-intervention Task 1	θ oscillatory power	0.66 ± 0.31	0.79 ± 0.23
	α oscillatory power	0.70 ± 0.25	0.92 ± 0.23
	β oscillatory power	0.48 ± 0.24	0.45 ± 0.19
	α/β ratio	0.23 ± 0.14	0.48 ± 0.28
Pre-intervention Task 3	θ oscillatory power	0.41 ± 0.59	0.11 ± 0.70
	α oscillatory power	0.59 ± 0.34	0.26 ± 0.47
	β oscillatory power	0.51 ± 0.35	0.25 ± 0.29
	α/β ratio	0.08 ± 0.49	0.01 ± 0.24
Pre-intervention Task 1	Number of answered questions	40.0 ± 0	39.3 ± 1.4
	Number of correct answers	39.9 ± 0.4	39.1 ± 1.4
	Number of incorrect answers	0.1 ± 0.4	0.1 ± 0.4
	Response time	33.0 ± 4.2	33.9 ± 5.5
Pre-intervention Task 3	Number of answered questions	36.7 ± 4.7	34.4 ± 5.1
	Number of correct answers	36.3 ± 4.5	34.3 ± 5.2
	Number of incorrect answers	0.4 ± 0.8	0.1 ± 0.4
	Response time	39.4 ± 1.0	40.0 ± 0

Data are presented as mean ± SD or *n*. Sex is presented as male/female. All participants were 21 years of age.

**Table 2:** EEG artifact exclusion rates and remaining duration of EEG data after artifact removal in the intervention and control groups.

		The Intervention Group AVG ± SE	The Control Group AVG ± SE	*p*	r
Rejection rate of epochs excluded due to artifacts, %	Pre-intervention Task 1	25.0 ± 11.6	24.2 ± 14.0	0.87	0.06
	Post-intervention Task 1	35.7 ± 15.5	14.6 ± 15.4	0.01	0.66
	Pre-intervention Task 3	25.3 ± 4.6	24.6 ± 6.5	0.87	0.06
	Post-intervention Task 3	19.9 ± 2.9	23.6 ± 5.4	0.12	0.40
EEG data retained after artifact rejection, seconds	Pre-intervention Task 1	30.7 ± 10.2	15.1 ± 7.4	0.004	0.70
	Post-intervention Task 1	28.4 ± 16.2	18.1 ± 10.9	0.19	0.35
	Pre-intervention Task 3	27.1 ± 4.1	27.3 ± 8.7	0.34	0.27
	Post-intervention Task 3	27.7 ± 6.1	32.3 ± 4.5	0.15	0.39

**Table 3:** Neural oscillations after intervention (αNFB), inter-group comparison.

		The Intervention Group AVG ± SE	The Control Group AVG ± SE	*p*	*p* (Corrected)	r	95% CI
Task 1 Pre–Post Difference	θ oscillatory power	0.11 ± 0.30	−0.35 ± 0.42	0.06	0.36	0.49	[−0.86, 0.00]
	α oscillatory power	0.03 ± 0.23	−0.39 ± 0.25	0.01	0.07	0.72	[−0.66, −0.12]
	β oscillatory power	−0.12 ± 0.12	−0.01 ± 0.19	0.18	0.90	0.34	[−0.08, 0.29]
	α/β ratio	0.14 ± 0.21	−0.38 ± 0.23	0.003	0.02	0.78	[−0.75, −0.26]
Task 3 Pre–Post Difference	θ oscillatory power	0.08 ± 0.75	0.12 ± 0.95	0.91	1.00	0.03	[−0.93, 0.98]
	α oscillatory power	−0.06 ± 0.28	0.12 ± 0.70	0.73	1.00	0.09	[−0.45, 0.69]
	β oscillatory power	−0.12 ± 0.19	−0.02 ± 0.36	0.73	1.00	0.09	[−0.17, 0.45]
	α/β ratio	0.07 ± 0.32	0.14 ± 0.39	1.00	1.00	0.00	[−0.29, 0.52]

AVG: average, SE: standard error. 95% CIs represent confidence intervals for the Hodges–Lehmann estimate of the median difference between groups.

**Table 4:** Task performance following intervention.

		The Intervention Group AVG ± SE	The Control Group AVG ± SE
Pre-intervention Task 1	Number of answered questions	40.0 ± 0.0	39.1 ± 1.5
	Number of correct answers	39.9 ± 0.4	39.1 ± 1.5
	Number of incorrect answers	0.1 ± 0.4	0.0 ± 0.0
	Response time	33.0 ± 4.2	35.1 ± 4.6
Post-intervention Task 1	Number of answered questions	40 ± 0.0	40 ± 0.0
	Number of correct answers	40 ± 0.0	40 ± 0.0
	Number of incorrect answers	0	0
	Response time	31.3 ± 4.9	31.0 ± 3.1
Pre-intervention Task 3	Number of answered questions	36.7 ± 4.7	33.6 ± 4.9
	Number of correct answers	36.3 ± 4.5	33.4 ± 5.0
	Number of incorrect answers	0.4 ± 0.8	0.1 ± 0.4
	Response time	39.4 ± 1.0	40.0 ± 0.0
Post-intervention Task 3	Number of answered questions	38.0 ± 2.8	39.6 ± 3.1
	Number of correct answers	38.0 ± 2.8	36.3 ± 3.1
	Number of incorrect answers	0	0
	Response time	39.1 ± 1.6	39.5 ± 0.8

AVG: average, SE: standard error.

**Table 5:** Effect of intervention on task performance, inter-group comparison.

		The Intervention Group AVG ± SE	The Control Group AVG ± SE	*p*	*p* (Corrected)	r	95% CI
Task 1 Pre–Post Difference	Number of answered questions	0.0 ± 0.0	0.8 ± 1.4	0.17	1.00	0.35	[0.00, 0.00]
	Number of correct answers	0.1 ± 0.4	0.9 ± 1.4	0.26	1.00	0.29	[0.00, 1.00]
	Number of incorrect answers	−0.1 ± 0.4	−0.1 ± 0.4	0.92	0.92	0.03	[0.00, 0.00]
	Response time	−1.7 ± 4.0	−3.9 ± 2.7	0.32	1.00	0.26	[−6.00, 2.00]
Task 3 Pre–Post Difference	Number of answered questions	1.3 ± 2.8	2.4 ± 2.3	0.21	1.00	0.33	[−1.00, 3.00]
	Number of correct answers	1.7 ± 2.6	2.5 ± 2.3	0.36	1.00	0.23	[−1.00, 3.00]
	Number of incorrect answers	−0.4 ± 0.8	−0.1 ± 0.4	0.41	1.00	0.21	[0.00, 1.00]
	Response time	−0.3 ± 1.4	−0.4 ± 0.8	0.82	1.00	0.06	[−1.00, 1.00]

AVG: average, SE: standard error. 95% CIs represent confidence intervals for the Hodges–Lehmann estimate of the median difference between groups.

## Data Availability

Data supporting the results of this study are available from the corresponding author, N.M., upon reasonable request. The data are not publicly available due to privacy and ethical restrictions related to human participant data.

## Abbreviations

The following abbreviations are used in this manuscript:

α-NFB	Alpha neurofeedback
EEG	Electroencephalogram
